# Effect of traction direction and pressure load on the palatal plate on retentive force

**DOI:** 10.1186/s12903-022-02313-z

**Published:** 2022-07-16

**Authors:** Kunihito Yamane, Yuji Sato, Junichi Furuya, Noboru Kitagawa, Naoya Ikemura, Osamu Shimodaira

**Affiliations:** Department of Geriatric Dentistry, Showa University School of Dentistry, Ota Ward, Tokyo, Japan

**Keywords:** Denture adhesive, Dry mouth, Palatal plate, Removable dentures, Retentive force

## Abstract

**Background:**

Recently, a denture adhesive that is easy to clean and contain moisturizing ingredients have been developed for patients with dry mouth. Although the retentive force produced by conventional denture adhesives and oral moisturizers have been compared on models, no study has reported their comparison in the oral cavity. In this study, we aimed to clarify the effects of different directions of traction and loads at the time of pressure contact on the retentive force on a palatal plate made from a dentulous jaw model.

**Methods:**

A palatal plate was fabricated with thermoplastic resin on a dentulous jaw model, and a loop-shaped traction device was attached to the centre of the palate. The test samples were a cream-type denture adhesive, a denture adhesive for dry mouth, an oral moisturizer, and a denture moisturizer. The specimens were applied to the inner surface of the plate, which was then mounted under vertical pressure. Then, the retentive force was measured, using a digital force gauge, while the plate was pulled in different directions and with different loads.

**Results:**

No significant difference in retentive force was observed in any of the test samples when the direction of traction was between 45° and 60°. The retentive force of the denture adhesive for dry mouth was significantly higher with a direction of traction of 90° than that of 45° or 60°. The retentive force when oral moisturizer was used was significantly higher in the 90° traction direction than in the 45° direction. There was no significant difference between a force of 4.0 kgf or 5.5 kgf when using a denture adhesive for dry mouth. Comparing the four load size conditions, the larger the load, the higher was the retentive force. Similar results were obtained for the cream-type denture adhesive and denture moisturizer. Significantly higher retentive force was observed for larger loads when oral moisturizer was used.

**Conclusions:**

The results suggest that the direction of traction and the pressure load affect the retentive force on a palatal plate.

## Background

While Japan's total population is declining, the proportion of older individuals in the population continues to increase. According to a report by the Statistics Bureau of the Ministry of Internal Affairs and Communications in FY2022, the aging rate is currently 29.3% and is expected to continue to rise [1]. As the older population increases, it is likely that more people will be wearing removable dentures. However, as individuals age, it is often difficult for them to maintain their dentures due to changes in their general condition and oral environment, such as multiple systemic diseases [2, 3], impaired movement of the mouth due to diseases [4, 5], progressive xerostomia caused by side-effects of drugs [6, 7], resorption of the alveolar ridge, and changes in the mandibular position [8, 9]. For older patients who have difficulty maintaining or stabilizing dentures, the use of denture adhesives may be effective [10].

On the other hand, denture adhesives are difficult to remove from the oral mucosa and denture surfaces after use. Residual denture adhesives can become a breeding ground for growth of oral bacteria and *Candida albicans*, causing denture stomatitis [11]. Additionally, they also increase the risk of aspiration pneumonia [12, 13]. Cream-type denture adhesives, which become sticky when they absorb water, do not easily enhance denture retention in a dry mouth. In addition, they are highly water absorbent and may exacerbate dry mouth. For patients with dry mouth, oral moisturizers that are washed off easily may be recommended as an alternative to denture adhesives [14–16]. However, many oral moisturizers contain artificial sweeteners, which may spoil the flavour of food. In addition, unlike denture adhesives, the safety of which has been confirmed by JIS and ISO standards, oral moisturizers are not originally indicated for use as denture adhesives.

Recently, a gel-type denture adhesive containing moisturizing ingredients has been developed for dry mouth [17]. Ohno et al. placed some test samples between two dried resin plates and measured its retentive force. The results showed that this denture adhesive provided significantly higher retention than a cream-type denture adhesive and an oral moisturizer [17]. Further, in a previous study that measured the retentive force achieved with denture adhesives on an edentulous jaw model over a period of time, the denture adhesive for dry mouth showed a significantly higher retentive force during the first 30 min of measurement than did the cream-type denture adhesive [18].

However, there is no report comparing the denture retentive force of denture adhesives for dry mouth and other denture adhesives in the oral cavity rather than on a model. Before measuring the retentive force of dentures in the oral cavity, it is necessary to examine the retentive force of palatal plates in healthy dentulous jawed subjects, whose oral moistness and salivary secretion are more stable than those of edentulous jawed subjects, and to set appropriate measurement conditions.

Therefore, the purpose of this study was to clarify the influence of the direction of traction and the load at the time of pressure contact on the plate, simulating the intraoral environment using a palatal plate made from a dentulous jaw model, and investigating the optimal measurement conditions in the oral cavity.

## Methods

### Test samples

The following four types of test samples were used in this study: cream-type denture adhesive (NP; New Poligrip® Sa; Glaxo Smith Kline, Tokyo, Japan), gel-type denture adhesive for dry mouth (DM; Pitatto Kaiteki Gel®; Nippon Shika Yakuhin Co., Ltd., Shimonoseki, Japan), gel-type oral moisturizer (BT; Biotene Oral Balance Jell®; T&K, Tokyo, Japan), and cream-type denture moisturizer (DW; Denture Wet®; DentCare Pvt., Ltd., Kerala, India).

### Palatal plate manufacturing

**Fig. 1:**
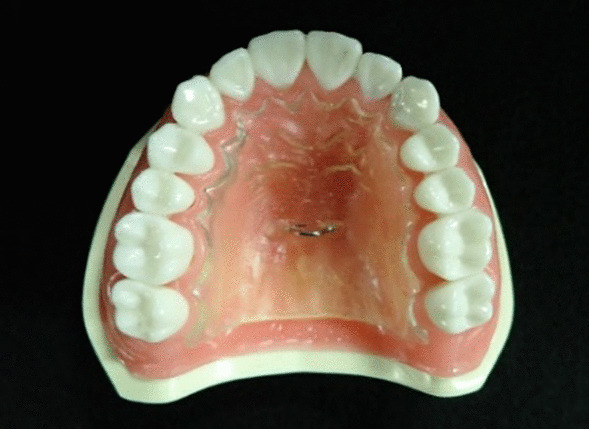
An upper dentulous jaw model and a palatal plate. A palatal plate was made from a 3.0 mm sheet of thermoplastic resin. A traction ring made from 0.9 mm diameter Co–Cr alloy wire was placed in the center of the palatal plate

A palatal plate was fabricated using a 3.0-mm sheet of thermoplastic resin (Erkodul®; Ercodent, Yokohama, Japan) from a working cast made by taking an impression of an upper dentulous jaw model. The plate was shaped such that it was 1.0 mm from the tooth cervices, and the rear edge was distal to the left and right second maxillary molars. A traction ring was made from 0.9-mm diameter Co-Cr alloy wire (SUN-COBALT CLASP-WIRE®; Dentsply Sirona, Tokyo, Japan). This was placed in the center of the palatal plate (on the intersection of the midline and the straight line connecting the central fossa of the bilateral first molars) (Fig. 1).

### Retentive force measuring device

**Fig. 2:**
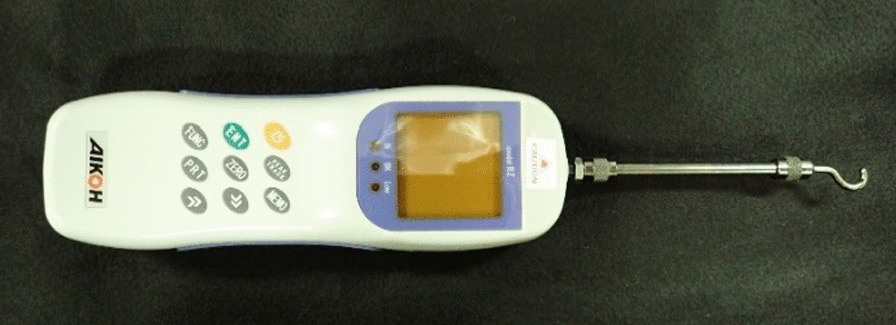
The measuring device (Digital force gauge RZ-5®;AIKOHENGINEERING, Tokyo, Japan). Measuring the retention of the palatal plate by hooking the tip of the device to a ring and applying traction

A digital force gauge (Digital Force Gauge RZ-5®; AIKOHENGINEERING, Tokyo, Japan) used as the measuring device (Fig. 2). We applied 2.5 g of the test sample to the inner surface of the palatal plate. Then, the plate was pressure perpendicular to the occlusal plane to the model for 10 s while the load was defined on a weighing instrument. A device indicating the direction of traction was mounted on the occlusal surface of the left first and second molar prosthesis of the model, and traction was exerted in a specified direction at a speed of 0.5 N/s using a digital force gauge. The force at which the palatal plate detached from the model was measured as the retentive force. The subsequent two experiments were conducted to investigate the influence of the direction of traction and load on the retentive force of the palatal plate. Only one palatal floor and one model should be prepared to avoid inconsistencies in the model's shape and other conditions for each measurement. The palatal plate and the model were washed after each measurement, and the following test sample was applied and measured without remnants from the previous sample.

### Experimental condition: effect of traction

**Fig. 3:**
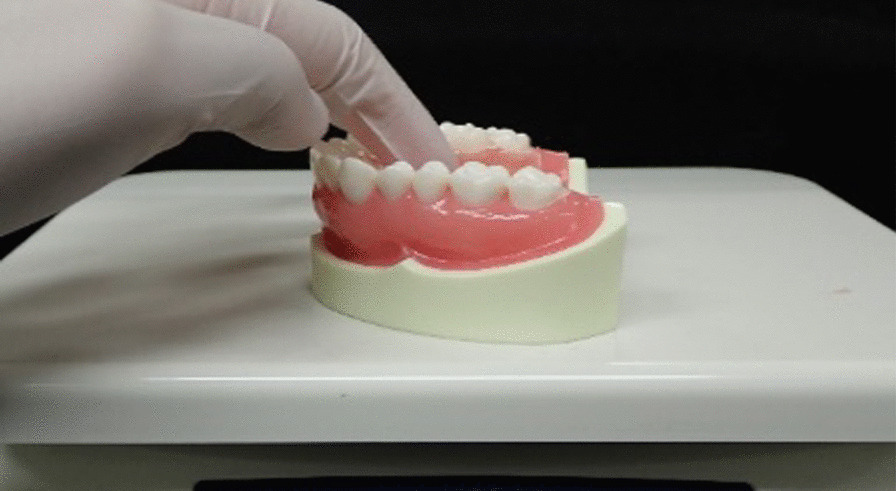
Pressure welding of the palatal floor to the model on the weighing instrument. The plate was pressure perpendicular to the occlusal plane to the model for 10 s while the load was defined on a weighing instrument

**Fig. 4:**
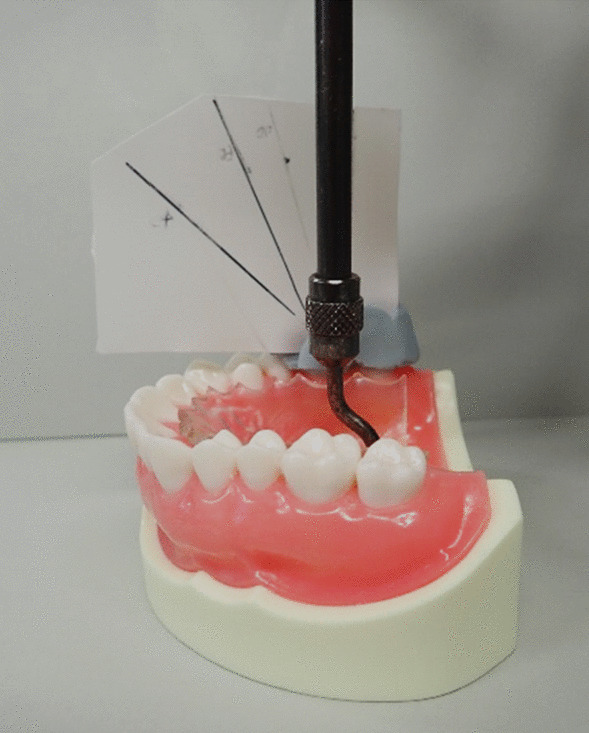
A device to guide the direction of traction. This device defines 45°, 60° and 90° directions to the occlusal plane

The palatal plate coated with the test sample was mounted onto the model, and a pressure load of 2.5 kgf was applied to the centre of the palatal plate on a weighing instrument (Fig. 3). Then, the digital force gauge was used to measure the retentive force by pulling in 45°, 60°, and 90° to the occlusal plane (Fig. 4). This was done six times (from pressure contact through traction to detachment) in each of the three directions. The first measurement was excluded, and the results from the second to the sixth measurements were used. The first measurement was excluded because it was not stable [18], and the average of the second through sixth measurements was obtained.

After six measurements, the sample was rinsed completely. Six consecutive measurements were taken three times, and three averages were obtained for the three sets of measurement. Finally, the average of the three averages was calculated.

### Experimental condition: effect of pressure load

The palatal plate with the test sample applied was mounted on the model, and pressure loads of 1.0 kgf, 2.5 kgf, 4.0 kgf, and 5.5 kgf were applied to the center of the palatal floor, on a weighing instrument. Then, a digital force gauge was used to measure the retentive force by pulling perpendicularly to the occlusal plane. The retentive force was measured for each of the four conditions of pressure load.

### Statistical analysis

SPSS Statistics 27.0 (IBM Corp, Armonk, NY, USA) was used for statistical analysis, and Tukey's method was used after one-way analysis of variance for multiple comparisons. All significance levels were set at 5%.

## Results

### Effect of traction direction

**Fig. 5:**
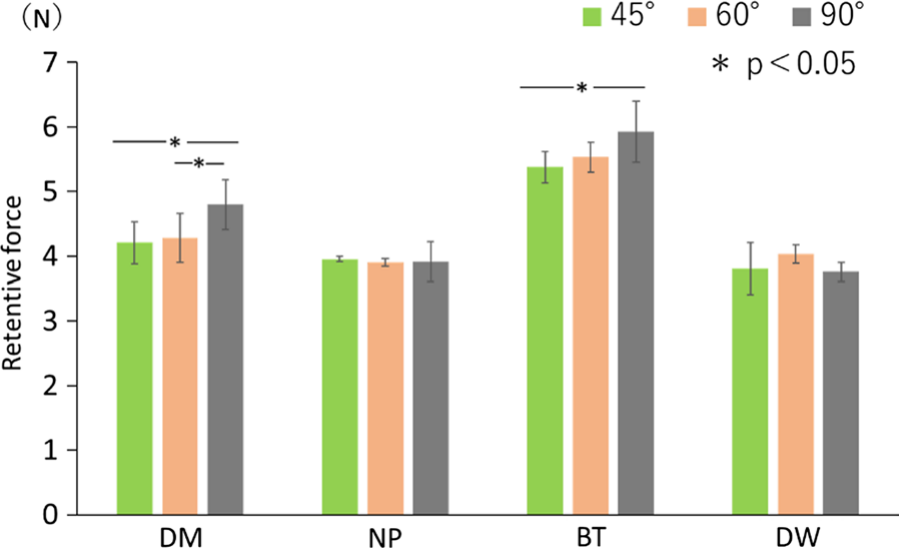
Retentive force measured on changing the direction of traction. The 90° traction direction showed the highest retention force with DM. When BT was used, the traction direction of 90° showed significantly higher retentive force than directions of 45° (*p* < 0.05). The effect of traction direction on the retentive force depends on the material. NP; New Poligrip® Sa; Glaxo Smith Kline, Tokyo, Japan (cream-type denture adhesive), DM; Pitatto Kaiteki Gel®; NISHIKA (gel-type denture adhesive for dry mouth), BT; Biotene Oral balance Jell®; T&K, Tokyo, Japan (gel-type oral moisturizer), DW; Denture Wet.®; DENTCARE (cream-type denture moisturizer)

There was no significant difference in retentive force between traction in the 45° and 60° directions for any of the test samples (*p* < 0.05; Table 1). The 90° traction direction showed the highest retention force with DM. When BT was used, the traction direction of 90° showed significantly higher retentive force than directions of 45° (*p* < 0.05) (Fig. 5).

**Table 1:** Retentive force measured on changing the direction of traction

	DM	NP	BT	DW
45°	4.20 ± 0.32	3.95 ± 0.04	5.37 ± 0.24	3.80 ± 0.40
60°	4.28 ± 0.37	3.90 ± 0.05	5.53 ± 0.23	4.03 ± 0.14
90°	4.79 ± 0.38	3.91 ± 0.31	5.92 ± 0.47	3.75 ± 0.14

### Effect of pressure load

**Fig. 6:**
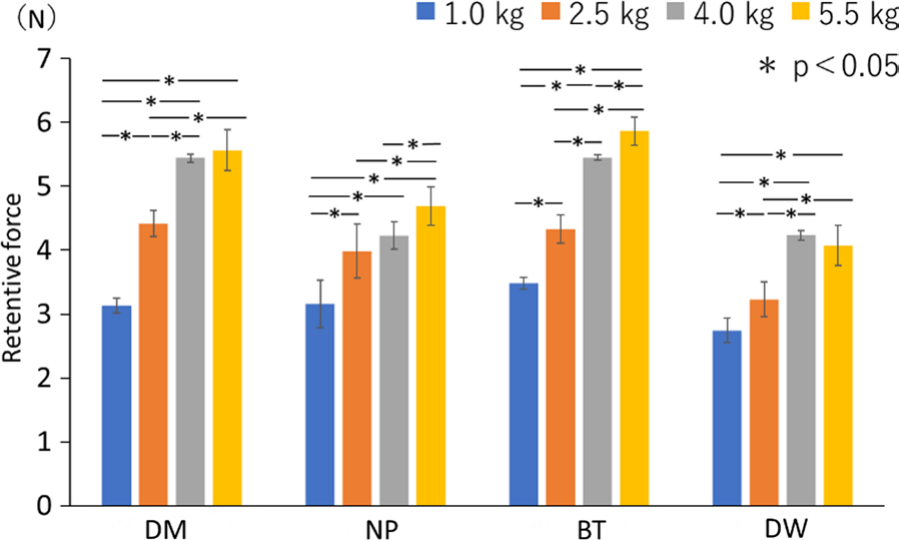
Retentive force measured on changing pressure loads. In all cases, the higher the pressure load, the higher tended to be the retentive force. The magnitude of the pressure load affects the retention force. NP; New Poligrip® Sa; Glaxo Smith Kline, Tokyo, Japan (cream-type denture adhesive), DM; Pitatto Kaiteki Gel®; NISHIKA (gel-type denture adhesive for dry mouth), BT; Biotene Oral balance Jell®; T&K, Tokyo, Japan (gel-type oral moisturizer), DW; Denture Wet®; DENTCARE (cream-type denture moisturizer)

In all cases, the higher the pressure load, the higher was the retentive force (Table 2). When DM was used, there was no significant difference between 4.0 and 5.5 kgf, while the retention force was significantly higher (*p* < 0.05) at higher pressure loads among the other loading conditions. Similar results were obtained when NP and DW were used. When BT was used, significantly higher retention was observed at a greater load (*p* < 0.05) (Fig. 6).

**Table 2:** Retentive force measured on changing pressure loads

	DM	NP	BT	DW
1.0 kg	3.13 ± 0.12	3.15 ± 0.36	3.48 ± 0.09	2.74 ± 0.19
2.5 kg	4.41 ± 0.20	3.98 ± 0.42	4.32 ± 0.22	3.23 ± 0.27
4.0 kg	5.44 ± 0.06	4.23 ± 0.21	5.44 ± 0.03	4.23 ± 0.07
5.5 kg	5.56 ± 0.31	4.68 ± 0.30	5.86 ± 0.22	4.07 ± 0.31

## Discussion

In this study, we aimed to clarify the effects of different directions of traction and different loads at the time of pressure contact on the retentive force on a palatal plate made from a dentulous jaw model. The retentive force did not differ between traction directions of 45° and 60° for any of the test samples, but the denture adhesive for dry mouth was significantly higher with a direction of traction of 90° than that of 45° and 60° The retentive force when oral moisturizer was used was significantly higher in the 90° traction direction than in the 45° direction. There was no significant difference between a force of 4.0 kgf or 5.5 kgf when using a denture adhesive for dry mouth, and the retentive force tended to be significantly higher at higher pressure loads under all loading conditions when any of the samples were used.

### Test samples

As in the previous study [18], there were four types of test samples: a cream-type denture adhesive, a gel-type denture adhesive for dry mouth whose main ingredient was water-soluble, an oral moisturizer, and a cream-type denture moisturizer with an oil-based ingredient. Yamagaki et al. reported that high-viscosity oral moisturizers have the same retentive force as denture adhesives [14]. In this study, BT, with high viscosity, was selected among oral moisturizers. DW is a substance applied to the denture surface to reduce friction against the oral mucosa during wearing.

The retentive force can be measured stably by filling the space between the model and the denture base’s mucosal surface with the test sample [18]. In preliminary experiments, the amount of sample applied that could fill the inner surface of the palatal plate without excess or deficiency was studied, and consequently 2.5 g was used as the set amount in this study.

### Palatal plate manufacturing

The palatal plate was designed to be 1 mm inward from the tooth cervices so that the floor area would be as wide as possible, without interfering with the prosthesis during traction. In addition, if the traction ring was higher than necessary, the backward detachment force during traction becomes large, and the retentive force may not be measured accurately. Therefore, we fabricated it with a height of 4 mm to match the tip of the retentive force measuring device.

### Effect of traction direction

Yamagaki et al. reported that a stable retentive force can be measured by setting the traction speed to 0.5 N/s when measuring the retentive force using a digital force gauge [14]. In this study, traction was also applied at 0.5 N/s. The denture adhesive for dry mouth and the oral moisturizer showed lower retentive force at 45° than at 90°, possibly due to the detachment from the back of the base caused by horizontal force arising from oblique traction. The cream-type denture adhesive and denture moisturizer, which have relatively low retentive force, were not affected by the direction of traction. Thus, the effect of traction direction on the retentive force depends on the material. Based on the above, an angle of 60° is considered to be optimal for actual intraoral measurements of retentive force, as this allows traction from outside the oral cavity, while minimizing the influence of the backward withdrawal force.

### Effect of pressure load

Bandai et al. examined the relationship between saliva viscosity and palatal plate retentive force and reported that 10 s of pressure contact stabilized the measured retentive force [19]. In the present study, we also measured the retentive force after 10 s of applying pressure. Kano et al. measured the thickness and adhesive force of denture adhesive between two resin plates and reported that the adhesive force increased with decreasing thickness [20]. In the present study, the higher the pressure load, the thinner was the thickness of the test sample between the plate and the model, and thus the higher was the retentive force. The magnitude of the pressure load affects the retention force. Therefore, if the pressure load is not specified in the oral cavity, the measured value of the retentive force may not be stable.

### Study limitation

It has been suggested that the direction of traction and the pressure load should be specified when measuring retentive force of dentures after applying denture adhesives or moisturizers. However, while it is easy to define in an experiment using a model, it is difficult to do so in the oral cavity. Therefore, it is necessary to devise a method to define the direction of traction and pressure load for use in such experiments in the oral cavity. In future, we plan to measure the retentive force of a palatal plate in the presence of denture adhesives and oral moisturizers in the mouths of dentulous individuals, using these defined measurement conditions.

## Conclusions

In this study, the direction of traction and the magnitude of pressure load were specified by attaching a device to the model to serve as a guide for the direction of traction on a scale, and measurements were made under various conditions. Our results suggest that the direction of traction and the magnitude of pressure load at the time of wearing the dentures affect the retentive force of the palatal floor.

## Data Availability

The datasets generated and/or analysed during the current study are not publicly available due [REASON WHY DATA ARE NOT PUBLIC] but are available from the corresponding author on reasonable request.

## Abbreviations

NP: Cream-type denture adhesive
DM: Gel-type denture adhesive for dry mouth
BT: Gel-type oral moisturizer
DW: Cream-type denture moisturizer
